# Influência da Localização Geográfica no Acesso às Terapias de Reperfusão e Mortalidade de Pacientes com IAMcSST em Sergipe: Registro VICTIM

**DOI:** 10.36660/abc.20200015

**Published:** 2021-07-15

**Authors:** Jeferson Cunha Oliveira, Guilherme José dos Santos Ferreira, Jussiely Cunha Oliveira, Ticiane Clair Remacre Munareto Lima, Ikaro Daniel de Carvalho Barreto, Laís Costa Souza Oliveira, Larissa Andreline Maia Arcelino, Antônio Carlos Sousa, José Augusto Soares Barreto-Filho

**Affiliations:** 1 Programa de Pós-Graduação em Ciências da SaúdeUniversidade Federal de SergipeSão CristóvãoSEBrasil Universidade Federal de Sergipe - Programa de Pós-Graduação em Ciências da Saúde da Universidade Federal de Sergipe , São Cristóvão , SE - Brasil; 2 Hospital PrimaveraAracajuSEBrasil Hospital Primavera , Aracaju , SE - Brasil; 3 Universidade Federal de SergipeSão CristóvãoSEBrasil Universidade Federal de Sergipe , São Cristóvão , SE - Brasil; 4 Universidade Federal Rural de PernambucoNúcleo de Pós-graduação em Biometria e Estatística AplicadaRecifePEBrasil Universidade Federal Rural de Pernambuco - Núcleo de Pós-graduação em Biometria e Estatística Aplicada , Recife , PE - Brasil; 5 Hospital UniversitárioUniversidade Federal de SergipeAracajuSEBrasil Hospital Universitário da Universidade Federal de Sergipe (HU-UFS), Aracaju , SE - Brasil; 6 Fundação São Lucas - Centro de Ensino e PesquisaAracajuSEBrasil Fundação São Lucas - Centro de Ensino e Pesquisa , Aracaju , SE – Brasil

**Keywords:** Doenças Cardiovasculares, Infarto do Miocárdio, Reperfusão Miocárdica, Mortalidade, Epidemiologia, Estudos de Corte Transversal

## Abstract

**Fundamento:**

A concentração de serviços de alta complexidade em Aracaju/SE pode proporcionar disparidade na qualidade assistencial para os pacientes do SUS com infarto agudo do miocárdio com supradesnivelamento do segmento ST (IAMcSST) cujos sintomas se iniciaram em outras regiões de saúde do estado.

**Objetivo:**

Avaliar disparidades no acesso às terapias de reperfusão e mortalidade em 30 dias, entre pacientes com IAMcSST, usuários do SUS, em cada uma das 7 regiões de saúde em Sergipe.

**Métodos:**

Foram avaliados 844 pacientes com IAMcSST no período de 2014 a 2018 atendidos pelo único hospital com capacidade de ofertar intervenção coronariana percutânea (ICP) primária para usuários do SUS no estado de Sergipe. Os pacientes foram divididos em 7 grupos de acordo com o local de início dos sintomas e obedecendo a divisão já existente das regiões de saúde do Estado. Para comparação entre grupos, foi considerada diferença significativa quando p < 0,05.

**Resultados:**

Do total de 844 pacientes vítimas de IAMcSST e transferidos ao hospital com ICP que atende pacientes do SUS, 386 pacientes (45,8%) realizaram angioplastia primária. A taxa média do uso de fibrinolítico foi de 2,6%, não havendo diferenças entre as regiões. O tempo médio total de chegada ao hospital com ICP foi de 21h55’ com mediana de 10h22’ (6h30’ – 22h52’). A mortalidade total em 30 dias foi 12,8%, mas sem diferenças entre as regiões, mesmo quando ajustada para idade e sexo.

**Conclusões:**

Este estudo revela que os fibrinolíticos são subutilizados em todo o estado e que existe um atraso significativo no acesso ao hospital com ICP, em todas as regiões de saúde de Sergipe.

## Introdução

As doenças cardiovasculares (DCV) representam a principal causa de morte no Brasil e no mundo. Dentro desse grupo, o infarto agudo do miocárdio com supradesnivelamento do segmento ST (IAMcSST) é responsável pela maior mortalidade na classe de doenças isquêmicas do coração, devido a sua severidade no prognóstico clínico. ^1^

Nesse contexto, o acesso precoce e a imediata reperfusão coronariana é o principal objetivo no tratamento do IAMcSST, por reduzir os resultados adversos e mortalidade. ^2 , 3^ Dentre as terapias de reperfusão a intervenção coronariana percutânea (ICP) primária e o uso de fibrinolítico são as principais estratégias terapêuticas. Entretanto a ICP, é considerada padrão ouro no tratamento, se realizada em menos de 12 horas do início dos sintomas e tem se mostrado superior ao fibrinolítico na redução da mortalidade, re-infarto e acidente vascular cerebral. ^4^

Estudo prévio desenvolvido por Oliveira et al., ^5^ mostrou que em Sergipe o tempo de chegada do início dos sintomas ao hospital com ICP (24,4 h ± 36,5 h), é o dobro de tempo do preconizado aos pacientes do SUS. As taxas de uso de fibrinolítico apesar de serem baixas nos serviços público e particular, são ainda menores nos pacientes do SUS e a mortalidade em 30 dias foi maior no SUS (11,9%) quando comparada aos pacientes do serviço privado (5,9%). Esses dados podem ser ainda piores quando comparados entre as regiões de saúde de Sergipe. ^5^

Ademais, baseado em princípios organizacionais do SUS, o estado é dividido em 7 regiões de saúde, porém, apesar da divisão, todos os hospitais com serviço de hemodinâmica estão situados em uma única região de saúde. E, para maior agravamento da situação, apenas um desses hospitais é referência cardiológica para os usuários do SUS e tem por característica não ser porta aberta.

Nesse contexto, o presente estudo objetiva avaliar as possíveis diferenças no tocante ao acesso às terapias de reperfusão e mortalidade em pacientes com IAMcSST atendidos exclusivamente pelo SUS entre as diferentes regiões de saúde de Sergipe.

## Materiais e métodos

Trata-se de estudo transversal, com abordagem quantitativa, com dados obtidos de dezembro de 2014 a março de 2018, que utilizou a base de dados do registro VICTIM (Via Crucis para Tratamento do Infarto do Miocárdio). Esta pesquisa foi aprovada pelo Comitê de Ética e Pesquisa da Universidade Federal de Sergipe, sob o número do parecer 483.749. Os dados foram coletados no único hospital do estado que conta com a disponibilidade de ICP primária pelo SUS. Este, por sua vez não possui sistema de porta-aberta, ou seja, os pacientes devem ser encaminhados de outros serviços de saúde já com o diagnóstico de IAMcSST confirmado.

A coleta foi realizada pelos pesquisadores mediante um instrumento próprio de pesquisa, o *case report form* (CRF), composto de variáveis sociodemográficas, condições clínicas à hospitalização, dados referentes ao tempo e caminho percorridos do início dos sintomas até o atendimento em hospital especializado, procedimento angiográfico e evolução dos pacientes durante a internação hospitalar após o IAM. As informações foram coletadas por meio de entrevista com o paciente ou acompanhante e dados do prontuário.

Foram incluídos pacientes de ambos os sexos, maiores de 18 anos, cujo início dos sintomas se deu dentro de território sergipano, com diagnóstico de IAMcSST confirmado pelo eletrocardiograma, de acordo com os critérios definidores propostos pela V Diretriz da Sociedade Brasileira de Cardiologia ^6^ , que tiveram atendimento fornecido exclusivamente pelo SUS e que assinaram o Termo de consentimento Livre e Esclarecido.

Utilizamos como critério de exclusão os indivíduos que: morreram antes da entrevista; que recusaram a participação na pesquisa; que tiveram início de sintomas fora de território sergipano; que receberam atendimento em rede privada; que não caracterizaram a Via Crucis, ou seja, aqueles pacientes que não percorreram o trajeto desde o início dos sintomas até a chegada ao hospital com capacidade de realizar ICP por terem apresentado o IAMcSST já dentro do hospital; aqueles cujo evento agudo de IAMcSST foi caracterizado como reinfarto (ocorreu dentro de 28 dias do infarto incidente); apresentaram mudança de diagnóstico durante a internação e os atendidos por convênio em hospital filantrópico.

**Figura 1:**
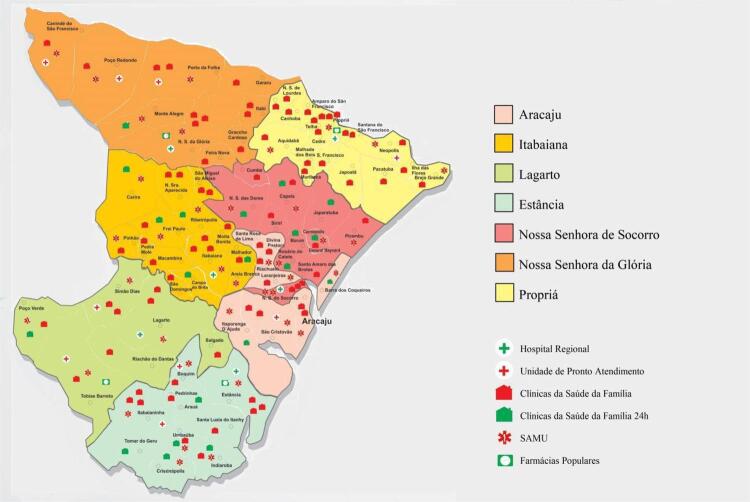
– Mapa de Sergipe e suas regiões de saúde. Fonte: (Secretaria De Estado de Saúde, 2016).

Obedecidos os critérios de inclusão do estudo, a alocação dos pacientes foi feita de forma consecutiva. Para a análise, os pacientes com IAMcSST foram divididos em 7 grupos, a partir da região de saúde de início de sintomas, são eles: 1. Aracaju, 2. Itabaiana, 3. Estância, 4. Lagarto, 5. Nossa Senhora do Socorro (Socorro), 6. Nossa Senhora da Glória (Glória), e 7. Propriá. Essas regiões de saúde foram delimitadas conforme a Deliberação nº 065/2012, de 18 de abril de 2012, que ratifica a divisão do território estadual de Sergipe em 7 regiões de saúde, determinando os municípios que compõem cada região ( Figura 1 ). O estado de Sergipe e seus 75 municípios, com base no último censo realizado pelo Instituto Brasileiro de Geografia e Estatística, possui população estimada pouco superior a dois milhões de habitantes, ^7^ que se dividem, na perspectiva do SUS, nessas 7 regiões de saúde. ^2^

## Análise Estatística

As variáveis categóricas foram descritas por meio de frequência absoluta e relativa. As associações foram testadas por meio do teste qui-quadrado com simulações de Monte-Carlo. As múltiplas comparações para as proporções foram testadas por meio de teste Z com correção de Bonferroni. As variáveis contínuas foram descritas por meio de mediana e intervalo interquartil devido a não aderência destas à distribuição normal avaliada pelo teste de Shapiro-Wilks. As diferenças nas medidas de tendência central foram testadas por meio do teste de Kruskal-Wallis. As múltiplas comparações para as medidas de tendência central foram testadas pelo teste de Kruskal-Wallis com correção de Bonferroni. Foram estimadas razões de chances brutas e ajustadas para a mortalidade geral em 30 dias por meio de regressão logística. O nível de significância adotado foi de 5% e o software utilizado foi o R Core Team 2019.

## Resultados

### Perfil Sociodemográfico

Foram analisados 844 pacientes dos quais, 294 (34,8%) eram da região de saúde Aracaju, 102 (12,1%) da região Itabaiana, 119 (14,1%) da região Estância, 122 (14,5%) da região Lagarto, 119 (14,1%) da região Socorro, 41 (4,85%) da região Glória e 47 (5,6%) da região Propriá.

A idade mediana total foi de 61 anos; dentre as regiões, Estância apresentou significativamente maior mediana de idade e a região Glória a menor. Em todas as regiões, houve prevalência do sexo masculino e da etnia não branca , havendo diferença entre a região Socorro quando comparada à Lagarto ou Glória (p = 0,02) ( Tabela 1 ).

**Tabela 1:** – Características sociodemográficas dos pacientes com IAMcSST por região de saúde*

Demografia	Todos	Aracaju	Itabaiana	Estância	Lagarto	Socorro	Glória	Propriá	p-valor
	(n=844)	(n=294)	(n=102)	(n=119)	(n=122)	(n=119)	(n=41)	(n=47)	
Idade - anos*	61 (52-70)	59 (50-68)	63,5 (50,75-71)	66 (55-74)§//	65 (57-73)§//	60 (51-67)	58 (51,5-69,5)	60 (54-67)	<0,001
**Sexo n (%)**									
Masculino	561 (66,5)	199 (67,7)	77 (75,5)	69 (58,0)	83 (68,0)	72 (60,5)	28 (68,3)	33 (70,2)	0,118
Feminino	283 (33,5)	95 (32,3)	25 (24,5)	50 (42,0)	39 (32,0)	47 (39,5)	13 (31,7)	14 (29,8)	
**Classe social† n (%)**									
A	3 (0,4)	3 (1,1)	0 (0)	0 (0)	0 (0)	0 (0)	0 (0)	0 (0)	0,025
B	17 (2,2)	12 (4,3)	1 (1,1)	1 (0,9)	2 (1,8)	0 (0)	1 (2,9)	0 (0)	
C	74 (9,4)	35 (12,5)	9 (9,8)	7 (6,3)	7 (6,2)	11 (9,9)	1 (2,9)	4 (8,9)	
D	240 (30,5)	97 (34,6)	26 (28,3)	30 (27)	26 (23)	39 (35,1)	12 (34,3)	10 (22,2)	
E	453 (57,6)	133 (47,5)	56 (60,9)	73 (65,8)§	78 (69)§	61 (55)	21 (60)	31 (68,9)	
**Etnia n (%)**									
Branco	248 (30)	85 (29,4)	36 (35,3)	31 (27,7)	45 (36,9)//	21 (18,3)	17 (42,5)//	13 (28,3)	0,020
Não Branco	578 (70)	204 (70,6)	66 (64,7)	81 (72,3)	77 (63,1)//	94 (81,7)	23 (57,5)//	33 (71,7)	
**Escolaridade n (%)**									
Nunca estudou	225 (26,7)	49 (16,7)	38 (37,3)§	41 (34,5)§	50 (41)§¶	28 (23,5)	12 (29,3)	7 (14,9) ‡	<0,001
Fundamental	457 (54,1)	153 (52)	48 (47,1)	70 (58,8)	61 (50)	67 (56,3)	24 (58,5)	34 (72,3)	
Médio	121 (14,3)	69 (23,5)	11 (10,8)	7 (5,9)§	8 (6,6)§	19 (16)	4 (9,8)	3 (6,4)	
Superior	32 (3,8)	20 (6,8)	3 (2,9)	1 (0,8)	3 (2,5)	3 (2,5)	0 (0)	2 (4,3)	
Pós-graduação	9 (1,1)	3 (1)	2 (2)	0 (0)	0 (0)	2 (1,7)	1 (2,4)	1 (2,1)	
**Estado Civil n (%)**									
Solteiro	115 (13,6)	45 (15,3)	10 (9,8)	15 (12,6)	18 (14,8)	18 (15,1)	4 (9,8)	5 (10,6)	0,050
Casado	415 (49,2)	139 (47,3)	52 (51)	51 (42,9)	72 (59)	53 (44,5)	23 (56,1)	25 (53,2)	
Mora com companheiro	130 (15,4)	42 (14,3)	13 (12,7)	26 (21,8)	15 (12,3)	20 (16,8)	9 (22)	5 (10,6)	
Divorciado	73 (8,6)	33 (11,2)	10 (9,8)	4 (3,4)	7 (5,7)	8 (6,7)	4 (9,8)	7 (14,9)	
Viúvo	111 (13,2)	35 (11,9)	17 (16,7)	23 (19,3)	10 (8,2)	20 (16,8)	1 (2,4)	5 (10,6)	

* Valores são mediana e intervalo interquartil. Classe social (IBGE) – A: > 20 salários mínimos, B: 10 – 20 salários mínimos, C: 4 – 10 salários mínimos, D: 2 – 4 salários mínimos, E: ≤ 2 salários mínimos. ‡ p < 0,05 quando comparado a Lagarto. § p < 0,05 quando comparado a Aracaju // p < 0,05 quando comparado a Socorro ¶ p < 0,05 quando comparado a Propriá ‡.

#### Características clínicas

Dentre os fatores de risco, a diabetes mellitus foi a única que apresentou variabilidade significativa, oscilando entre 17,1% em Glória e 42,6% em Propriá (p = 0,026). Os demais fatores de risco apresentaram prevalências semelhantes entre os grupos ( Tabela 2 ).

**Tabela 2:** – Características clínicas dos pacientes com IAMcSST por região de saúde*

Características Clínicas	Todos		Aracaju	Itabaiana	Estância	Lagarto	Socorro	Glória	Propriá	p-valor
	(n=844)		(n=294)	(n=102)	(n=119)	(n=122)		(n=41)	(n=47)	
**Risco Cardiovascular n (%)**							**(n=119)**			
Hipertensão	530	(62,8)	176 (59,9)	61 (59,8)	72 (60,5)	76 (62,3)	86 (72,3)	26 (63,4)	33 (70,2)	0,282
Diabetes	275	(32,6)	104 (35,4)	24 (23,5)	46 (38,7)	35 (28,7)	39 (32,8)	7 (17,1)	20 (42,6)	0,026
Dislipidemia	314	(37,2)	116 (39,5)	33 (32,4)	46 (38,7)	45 (36,9)	39 (32,8)	15 (36,6)	20 (42,6)	0,762
Tabagismo	282	(33,4)	102 (34,7)	37 (36,3)	32 (26,9)	32 (26,2)	45 (37,8)	20 (48,8)	14 (29,8)	0,078
**Número de Fatores de Risco n (%)**										
0	100	(11,8)	38 (12,9)	14 (13,7)	15 (12,6)	20 (16,4)	6 (5)	3 (7,3)	4 (8,5)	0,255
1	279	(33,1)	90 (30,6)	36 (35,3)	39 (32,8)	44 (36,1)	42 (35,3)	15 (36,6)	13 (27,7)	
2	297	(35,2)	100 (34)	38 (37,3)	40 (33,6)	35 (28,7)	50 (42)	18 (43,9)	16 (34)	
≥ 3	168	(19,9)	66 (22,4)	14 (13,7)	25 (21)	23 (18,9)	21 (17,6)	5 (12,2)	14 (29,8)	
Pressão arterial sistêmica mmHg *†										
Pressão arterial sistólica	140 (123-160)		145 (130-160,75)	140 (123,7-157,5)	135 (120-160)	140 (120,75- 157)	140 (120,75-160)	130 (120-147)¶	140 (120-160)¶	0,016
Pressão arterial diastólica	83 (75-96)		87,5 (78-99,25)	87 (76,75-93)	80 (70-99)	80 (71,75-90)	87 (77-98,5)	80(70-87,5)	80 (71-90)	0,007
**Killip‡**										
I	716	(85,3)	245 (83,9)	92 (92)	101 (84,9)	102 (84,3)	104 (87,4)	35 (85,4)	37 (78,7)	0,557
II	92 (11)		35 (12)	7 (7)	14 (11,8)	14 (11,6)	10 (8,4)	5 (12,2)	7 (14,9)	
III	18	(2,1)	7 (2,4)	1 (1)	3 (2,5)	2 (1,7)	1 (0,8)	1 (2,4)	3 (6,4)	
IV	13	(1,5)	5 (1,7)	0 (0)	1 (0,8)	3 (2,5)	4 (3,4)	0 (0)	0 (0)	
**GRACE score§**										
< 140 (baixo risco)	392	(50,2)	154 (58,1)#	41 (45,1)	51 (45,1)	42 (35,6)	63 (56,3)#	23 (56,1)	18 (43,9)	0,001
> 140 (alto risco)	389	(49,8)	111 (41,9)#	50 (54,9)	62 (54,9)	76 (64,4)	49 (43,8)#	18 (43,9)	23 (56,1)	
**Eletrocardiograma//**										
Anterior	527	(62,5)	198 (67,3)	63 (61,8)	71 (59,7)	74 (60,7)	68 (57,6)	22 (53,7)	31 (66)	0,384
Não-anterior	316	(37,5)	96 (32,7)	39 (38,2)	48 (40,3)	48 (39,3)	50 (42,4)	19 (46,3)	16 (34)	

* Valores são mediana e intervalo interquartil. † valor do primeiro registro (em milímetros de mercúrio) documentado no prontuário a partir da admissão hospitalar. No momento da internação - Classe I: ausência de estertores sobre os campos pulmonares e ausência de terceira bulha,Classe II: estertores em mais de 50% das áreas do pulmão ou a presença de terceira bulha, Classe III: estertores em mais de 50% dos campos pulmonares, Classe IV: choque. Variando de 0 a 263 calculado com o os dados de idade, frequência cardíaca, pressão arterial sistólica, creatinina e Killip registrados na admissão hospitalar. // Anterior: em derivações V1-V4, não anterior: em nenhuma das derivações V1-V4. ¶ p < 0,05 quando comparado com Aracaju. # p < 0,05 quando comparado a Lagarto

Quanto aos valores de pressão arterial sistêmica admissionais no hospital com ICP ( Tabela 2 ), a pressão arterial sistólica teve mediana total de 140 mmHg, apresentando maior valor na região Aracaju e menor em Nossa Senhora da Glória, com diferenças quando comparadas entre si (p = 0,016). Já a pressão arterial diastólica, atingiu mediana total de 83 mmHg apresentando maior mediana em Aracaju, Nossa Senhora do Socorro e Itabaiana e menor mediana em Nossa Senhora da Glória, Estância, Lagarto e Propriá (p = 0,007).

A região de Lagarto apresentou a maior taxa de pacientes considerados de alto risco de mortalidade pelo GRACE score ( Tabela 2 ) (64,4%), enquanto Aracaju apresentou a menor (41,9%), sendo possível observar diferença quando comparadas as regiões de Aracaju e Nossa Senhora da Glória em relação à Lagarto (p = 0,001). Do total de pacientes, 85,3% apresentaram Killip I e 62,5% dos IAMcSST foram de parede anterior. Esse padrão de comportamento se repetiu entre as regiões.

#### Reperfusão coronariana

A taxa de ICP primária total foi de 45,8%, sendo a região Aracaju com a maior taxa (51,9%) e Glória com a menor (17,1%), notando-se diferença quando comparada à Aracaju ou Itabaiana (p = 0,03). Do total, 25,1% dos pacientes do estudo não realizaram ICP e a taxa de uso do fibrinolítico total foi de 2,6% ( Tabela 3 ).

**Tabela 3:** – Procedimentos realizados em pacientes com IAMcSST por regiões de Saúde*

Procedimento‡	Todos	Aracaju	Itabaiana	Estância	Lagarto	Socorro	Glória	Propriá	p-valor
	(n=844)	(n=294)	(n=102)	(n=119)	(n=122)	(n=119)	(n=41)	(n=47)	
Fibrinolítico n (%)	22 (2,6)	3 (1)	3 (2,9)	5 (4,2)	5 (4,1)	4 (3,4)	1 (2,4)	1 (2,1)	0,477
ICP primária n (%)	386 (45,8)	151 (51,4)†	51 (50)†	55 (46,6)†	53 (43,4)	51 (42,9)	7 (17,1)	18 (38,3)	0,003
ICP não primária n (%)	275 (32,6)	84 (28,6)	31 (30,4)	44 (37)	37 (30,3)	43 (36,1)	21 (51,2)	15 (31,9)	0,092
Não realizou ICP n (%)	212 (25,1)	67 (22,8)	23 (22,5)	29 (24,6)	36 (29,5)	29 (24,4)	13 (31,7)	15 (31,9)	0,595
Cirurgia de revascularização miocárdica n (%)	24 (2,8)	7 (2,4)	5 (4,9)	3 (2,5)	2 (1,6)	2 (1,7)	3 (7,3)	2 (4,3)	0,383

*‡ ICP: intervenção coronariana percutânea. † p < 0,05 quando comparado a Glória.*

O tempo médio entre o início dos sintomas e a chegada em hospital com ICP foi de 21h55’ com mediana de 10h22’ (6h30’ – 22h52’), sendo Glória a apresentar o maior atraso, e Aracaju o menor. Foi registrada diferença estatística quando comparadas regiões de Glória e Socorro em relação à Aracaju (p = 0,001). Dos períodos que compõem todo esse curso temporal, o tempo decorrido entre a chegada no hospital sem ICP até a chegada ao hospital com ICP foi o mais impactante, registrando um tempo mediano de 7h37’. Nesse quesito, a região de maior atraso foi Glória, e a de menor Aracaju, sendo possível observar diferenças quando comparadas as regiões de Lagarto e Glória frente a Aracaju ( Tabela 4 ).

**Tabela 4:** – Curso Temporal e geográfico entre o início dos sintomas até a chegada no hospital com ICP por região de saúde*

	Todos	Aracaju	Itabaiana	Estância	Lagarto	Socorro	Glória	Propriá	p-Valor
	(n=844)	(n=294)	(n=102)	(n=119)	(n=122)	(n=119)	(n=41)	(n=47)	
Linha do Tempo *†									
Tempo A	30’ (10’-2h)	30’ (10’-2h37’)	30’ (15’-2h)	30’ (10’-2h)	30’ (15’-2h)	30’ (10’-2h)	30’ (17’-4h)	1h (10’-2h)	0,808
Tempo B	30’ (20’-1h)	30’ (15’-48’)	30’ (20’-1h)	30’ (20’-1h)	30’ (15’-50’)	30’ (18’-1h)	30’ (20’-1h)	30’ (20’-1h)	0,038
Tempo C	7h37’ (4h51’-18h)	6h30’ (3h30’-15h40’)	7h30’ (4h53’-13h)	7h50’ (5h20’-11h16’)	8h34’ (5h28’-21h25’) ‡	8h17’ (4h40’-28h15’)	12h40’ (6h45’-26h56’)‡	8h57’ (6h35’-14h30’)	<0,001
Tempo D	10h22’ (6h30’-22h52’)	9h (5h7’-21h32’)	10h (6h9’-15h47’)	10h30’ (7h10’-19h45’)	11h31’ (7h3’-25h20’)	12h40’ (6h30’-32h)‡	16h (8h30’-32h01’)‡	12h27’ (7h50’-18h08’)	0,001
**Nº de unidades percorridas**									
0	14 (1,7)	9 (3,1)	1 (1)	1 (0,8)	2 (1,6)	1 (0,8)	0 (0)	0 (0)	0,483
1	685 (81,2)	248 (84,4)	85 (83,3)	91 (76,5)	100 (82)	93 (78,2)	31 (75,6)	37 (78,7)	
2	125 (14,8)	32 (10,9)	14 (13,7)	24 (20,2)	17 (13,9)	20 (16,8)	9 (22)	9 (19,1)	
≥ 3	20 (2,4)	5 (1,7)	2 (2)	3 (2,5)	3 (2,5)	5 (4,2)	1 (2,4)	1 (2,1)	

* Valores são mediana e intervalo interquartil. † Tempo A: início dos sintomas à decisão de chamar socorro, Tempo B: decisão de chamar socorro ao hospital sem ICP, Tempo C: Chegada do hospital sem ICP ao hospital com ICP, Tempo D: Início dos sintomas ao hospital com ICP. ‡ p < 0,05 quando comparado com Aracaju.

Em relação ao número de instituições percorridas, até o hospital com ICP, a grande maioria dos pacientes (81,2%) passaram por pelo menos uma instituição antes do hospital com ICP. Cerca de 2,4% dos pacientes passaram por pelo menos três instituições antes do hospital com ICP, enquanto que apenas 1,7% tiveram acesso direto a este hospital ( Tabela 4 ).

#### Mortalidade

Ao avaliar a mortalidade em 30 dias ( Tabela 5 ), observou-se que a região de Estância obteve a maior taxa (18,6%) e Nossa Senhora da Glória a menor (7,5%) (p = 0,03). Quando ajustada para idade e sexo, adotando a região Aracaju como grupo controle, não foram observadas diferenças estatísticas.

**Tabela 5:** – Razão de chances da mortalidade em 30 dias nos pacientes com IAMcSST por região de saúde ajustada por idade e sexo

Regiões de saúde	Mortalidade N (%)	Mortalidade não ajustada OR (IC95%)	Mortalidade ajustada OR (IC95%)	p-valor
Aracaju*	27 (9,4) 27 (9,4)	1	1	
Itabaiana	9 (9,2)	0,98 (0,44-2,16)	0,93 (0,32-2,70)	0,900
Estância	22 (18,6)	2,22 (1,20-4,08)	1,70 (0,74-3,92)	0,214
Lagarto	22 (18,5)	2,19 (1,19-4,03)	2,07 (0,93-4,61)	0,074
Socorro	15 (12,7)	1,41 (0,72-2,75)	2,04 (0,86-4,87)	0,106
Glória	3 (7,5)	0,78 (0,23-4,68)	0,66 (0,13-3,48)	0,625
Propriá	8 (17)	1,98 (0,84-4,68)	1,93 (0,61-6,11)	0,263

*Representa o grupo controle.

Discussão

Três principais achados marcaram expressivamente os resultados desse estudo. O primeiro aponta grandes atrasos na chegada dos pacientes com IAMcSST no hospital com ICP, independentemente do local de início dos sintomas. O segundo atesta disparidades no uso das terapias de reperfusão entre pacientes nas diferentes regiões de saúde. O terceiro mostra a influência regional na mortalidade. Tais achados demonstram que há necessidade urgente de melhorarias na qualidade assistencial para pacientes com IAMcSST em todo o estado de Sergipe.

Apesar do maior conhecimento a respeito das metas terapêuticas no manejo do IAMcSST, a reprodutibilidade desses alvos ainda é tarefa difícil, principalmente no âmbito da saúde pública no Brasil. ^8 , 9^ Os pacientes que iniciam o quadro de IAMcSST no estado de Sergipe possuem uma taxa de mortalidade em 30 dias longe do que se considera desejável. Um registro realizado pela Sociedade Europeia de Cardiologia, incluindo países, mostrou mortalidade intra-hospitalar variando de 3,1% a 6,1%. ^10^ Um ensaio francês, executado entre os anos de 1995 a 2010, constatou uma queda na mortalidade em 30 dias de 13,7% para 4,4%. Esse decréscimo se deve a múltiplos fatores dentro da assistência ao paciente com IAMcSST, como o aumento na quantidade de unidades de terapia intensiva móveis, aumento no número de campanhas públicas informativas a respeito dos sintomas relacionados ao agravo e menor atraso tanto na chegada em hospital capacitado, quanto na decisão de procurar socorro. ^11^

Concomitantemente, ambas as regiões com piores taxas apresentavam as maiores faixas etárias médias. Apesar da redução da mortalidade por síndromes coronarianas agudas em todas as idades, sabe-se que pacientes com maior faixa etária apresentam pior prognóstico frente aos mais jovens, tanto por apresentarem maior número de comorbidades quanto por menor uso das terapias de reperfusão e medicamentosas. ^3 , 12 , 13^

Antes de chegarem ao hospital com capacidade de realizar ICP, alguns pacientes necessitaram passar primeiramente por pelo menos uma unidade de saúde sem este serviço até serem transferidos para o hospital especializado. Esses dados também foram analisados neste estudo e obteve-se como resultado que a maioria dos pacientes passou por pelo um hospital antes de chegar ao hospital com capacidade para realizar ICP, fato este que já era esperado, uma vez que o único hospital público com capacidade de realizar ICP não possui atendimento de porta aberta. Alguns pacientes que tem acesso direto ao hospital com ICP foram regulados e encaminhados pelo SAMU.

O período entre o início dos sintomas e o acesso a um serviço de hemodinâmica apresenta papel decisivo no prognóstico do paciente. ^14^ Em Sergipe, nesse período o tempo dispendido foi em média de 21 horas e 55 minutos com mediana de 10h22’ (6h30’ – 22h52’), beirando o dobro da janela de 12 horas estabelecidas pelas diretrizes nacionais e internacionais. Avaliando numa perspectiva regional, esse intervalo médio alcançou valores de 26 horas e 24 minutos e 26 horas e 10 minutos nas regiões de Socorro e Glória, respectivamente, sendo mais curto em Estância, com 16 horas e 22 minutos. No entanto, apesar dessa discrepância, só foi possível observar diferenças estatísticas quando comparadas Socorro e Glória à Aracaju. Vale ressaltar que o município mais distante da capital está a cerca de 3 horas de distância.

No estado de Sergipe, o curso temporal vivido por pacientes com IAMcSST desde o início dos sintomas até o acesso a hospital capacitado sofre enorme impacto do período inter-hospitalar, constituindo aproximadamente 87% de todo o processo. Nessa perspectiva, esses resultados não correspondem à realidade geográfica de Sergipe, que, considerado o menor estado da Federação, é necessário em torno de 4 horas de carro para ir de uma extremidade à outra do estado. Além disso, a região de Socorro, apesar da relativa proximidade à região Aracaju frente às demais, paradoxalmente, apresenta o maior desses hiatos com 23 horas e 15 minutos.

Vários elementos podem se atrelar ao prolongamento desse intervalo, podendo variar desde atrasos no diagnóstico da doença até ineficiência nos métodos de transporte entre as instituições assistentes. ^1 , 15^ Um dado que merece destaque, é a impossibilidade de encaminhamento dos pacientes diretamente ao hospital habilitado. Em Sergipe, o único hospital capaz de ofertar tratamento definitivo para pacientes portadores de IAMcSST do SUS apenas acolhe pacientes mediante encaminhamento realizado por outra instituição, desde que já diagnosticados.

Um estudo realizado no Estado da Carolina do Norte, no período entre os anos de 2008 e 2010 avaliou 1.288 pacientes com diagnóstico de IAMcSST, dividindo-os em dois grupos; aqueles que eram transferidos diretamente para Hospitais com ICP independente da distância e aqueles que eram transportados para o hospital mais próximo sem ICP. Nessa comparação, 46,5% dos pacientes transferidos diretamente para um centro com serviço de hemodinâmica chegavam dentro dos 90 minutos após primeiro contato médico, enquanto no outro grupo apenas 21,5% dos pacientes alcançavam um centro de hemodinâmica dentro dos 120 minutos após primeiro contato médico. ^16^

Considerando-se a grande dificuldade de acesso no único hospital com capacidade de realizar ICP primária, menos da metade dos pacientes chegam na janela das 12 h do início dos sintomas, o expressivo subuso do fibrinolítico no estado de Sergipe pode ser considerado um indicador de péssima prática assistencial e contribuinte para a elevada mortalidade observada no nosso registro. A taxa média de uso de fibrinolítico de 2,6% difere bastante de outros registros ^17 , 18^ nacionais e internacionais e, no cenário de dificuldade de acesso ao único hospital com capacidade de realização de ICP primária, expõe de forma inequívoca a grande fragilidade e ineficiência do atendimento do IAMcSST no nosso estado. ^4^ Esses nossos achados poderiam ser até mais críticos, se a população estudada fosse composta de todos os pacientes com IAMcSST do estado, ou seja, os que ficaram em hospitais primários e secundários, sem acesso ao hospital terciário. Municiar a rede de hospitais regionais com capacidade de realizar trombólise no cenário poderá aumentar a taxa total de pacientes reperfundidos e reduzir a mortalidade nesse cenário.

Apesar de a angioplastia primária ser o tratamento de escolha para esses pacientes. No presente estudo, apenas 45,8% dos pacientes foram submetidos a essa terapêutica, atingindo valores mais gritantes na perspectiva da região Glória, com 17,1%. Estas taxas estão abaixo do que se encontra em outros estudos. ^19 - 22^ Apesar da variabilidade entre as regiões, só foi possível observar diferença estatística quando comparada a região de Lagarto a Aracaju.

As discrepâncias encontradas tanto na taxa de ICP, quanto no tempo de acesso a esse método, se justificam pelas mesmas falhas da rede assistencial. Um estudo conduzido no Reino Unido, mostrou que, na segunda metade de 2011, 94% dos pacientes com IAMcSST foram tratados por meio da ICP, aumento significativo quando comparado a taxa de 46% no ano de 2008. Dentre os elementos responsáveis nessa progressão estão: transporte de pacientes diretamente para centros com ICP, treinamento profissional para diagnóstico pré-hospitalar, coleta de dados a respeito da qualidade da assistência, e por último, criação de políticas nacionais no sentido de facilitar o acesso à rede de saúde. ^23^

Um outro estudo realizado no Reino Unido utilizando dados da Inglaterra e do País de Gales, contando com um total de 228 hospitais e uma amostragem de 34.722 pacientes com IAMcSST, demonstrou que o uso de aspirina na admissão e a trombólise fora de ambiente hospitalar são os mais fortes preditores de sobrevivência intra-hospitalar. Além disso, fatores como frequência cardíaca e pressão arterial sistólica admissionais também impactam na mortalidade em 30 dias por IAMcSST. ^3^

## Limitações

Tendo em vista a precariedade de registros bem documentados em algumas das regiões, a obtenção dos dados foi complementada por meio de entrevista e parte destes foram auto referidos, o que pode possibilitar imprecisões nas medidas temporais. Também vale notificar que, muitos usuários do SUS desconhecem seu estado de saúde prévio, dificultando a mensuração precisa da prevalência das comorbidades, esse fato dificulta o ajuste de risco entre as diferentes populações das regiões investigadas. Por último, o presente estudo restringiu a coleta de dados ao hospital com ICP, por ser a única referência em tratamento de IAMcSST no estado. Esse fato limita os resultados aos pacientes que tiveram acesso ao centro de referência. Entretanto, destacamos que por limitarmos a população ao centro de referência, o cenário observado deve representar a melhor qualidade assistencial praticada pelo SUS no estado de Sergipe.

## Conclusão

O registro VICTIM flagrou gritante hipossuficiência quanto ao acesso a ICP no estado, registrando uma janela temporal de acesso primário quase o dobro do que se considera limítrofe, sendo ainda pior em algumas sub-regiões. Ademais, existe uma subutilização marcante no uso dos fibrinolíticos, como terapia alternativa de reperfusão para o IAMcSST, em todas as regiões. Altas taxas de mortalidade persistem, apesar dos progressos terapêuticos da ciência cardiovascular na era da reperfusão miocárdica. Em conjunto, nossos dados demonstram uma grande ineficiência do SUS, no quesito assistência ao paciente com IAMcSST, no estado de Sergipe. Nossos resultados deveriam ser investigados em outros estados e regiões do país para avaliar se os precários indicadores observados no presente estudo são peculiares de Sergipe, da região inserida ou representam o “padrão” do atendimento do sistema de saúde pública no Brasil.
