# Supplementary material for: Influência da Localização Geográfica no Acesso às Terapias de Reperfusão e Mortalidade de Pacientes com IAMcSST em Sergipe: Registro VICTIM
Source: Arq Bras Cardiol. 2021 Jul 15;117(1):120–9. [Article in Portuguese] doi: 10.36660/abc.20200015 (PMC8294745; doi:10.36660/abc.20200015)
Supplement: Translation

# Translation: Influence of Geographical Location on Access to Reperfusion Therapies and Mortality of Patients with STEMI in Sergipe: VICTIM Register

**Authors:** Jeferson Cunha Oliveira, Guilherme José dos Santos Ferreira, Jussiely Cunha Oliveira, Ticiane Clair Remacre Munareto Lima, Ikaro Daniel de Carvalho Barreto, Laís Costa Souza Oliveira, Larissa Andreline Maia Arcelino, Antônio Carlos Sousa, José Augusto Soares Barreto-Filho

**Affiliations:** 1 Brazil Universidade Federal de Sergipe - Programa de Pós-Graduação em Ciências da Saúde da Universidade Federal de Sergipe, São Cristóvão, SE - Brazil; 2 Brazil Hospital Primavera, Aracaju, SE - Brazil; 3 Brazil Universidade Federal de Sergipe, São Cristóvão, SE - Brazil; 4 Brazil Universidade Federal Rural de Pernambuco - Núcleo de Pós-graduação em Biometria e Estatística Aplicada, Recife, PE - Brazil; 5 Brazil Hospital Universitário da Universidade Federal de Sergipe (HU-UFS), Aracaju, SE - Brazil; 6 Brazil Fundação São Lucas - Centro de Ensino e Pesquisa, Aracaju, SE – Brazil

**Keywords:** Cardiovascular Diseases, Myocardial Infarction, Myocardial Reperfusion, Mortality, Epidemiology, Cross-Sectional Studies

## Abstract

**Background:**

The concentration of high-complexity services in Aracaju, Sergipe can impose certain disparity in the quality of care for the patients with ST-segment elevation acute myocardial infarction (STEMI) (STEMI) who receive care from Brazil’s Unified Health System (SUS, acronym in Portuguese) and whose symptoms started in other health regions of the state.

**Objective:**

To evaluate disparities in access to reperfusion therapies and 30-day mortality, among patients with STEMI, who were users of SUS, in each of the 7 health regions of Sergipe.

**Methods:**

A total of 844 patients with STEMI in the period from 2014 to 2018, assisted by the only hospital with the capacity to offer primary percutaneous coronary intervention (PPCI) to SUS users in the state of Sergipe, were evaluated. The patients were divided into 7 groups according to the location at the onset of symptoms, following the existing division of health regions in the state. For comparison between groups, a significant difference was considered when p < 0.05.

**Results:**

Of the total of 844 patients suffering from STEMI who were transferred to the hospital with PPCI that serves SUS patients, 386 patients (45.8%) underwent primary angioplasty. The mean rate of fibrinolytic use was 2.6%, with no differences between the regions. The mean total time of arrival to the hospital with PPCI was 21 hours and 55 minutes, with a median of 10 hours and 22 minutes (6 hours and 30 minutes to 22 hours and 52 minutes). Total 30-day mortality was 12.8%, but without differences between the regions, even when adjusted for age and sex.

**Conclusions:**

This study reveals that fibrinolytics are underused throughout the state and that there is a significant delay in access to the hospital with PPCI, in all health regions of Sergipe.

## Introduction

Cardiovascular diseases (CVDs) represent the main cause of death in Brazil and worldwide. Within this group, ST-segment elevation acute myocardial infarction (STEMI) is responsible for the highest mortality in the ischemic heart disease class, due to its severity in clinical prognosis. ^1^

In this context, early access and immediate coronary reperfusion is the main objective in the treatment of STEMI, because they reduce adverse outcomes and mortality. ^2 , 3^ Among the reperfusion therapies, primary percutaneous coronary intervention (PPCI) and the use of fibrinolytics are the main therapeutic strategies. However, PPCI is considered the gold standard in treatment, if performed within fewer than 12 hours after the onset of symptoms, and it has been shown to be superior to fibrinolytics in reducing mortality, reinfarction and stroke. ^4^

A previous study developed by Oliveira et al. ^5^ showed that, in Sergipe, the time from the onset of symptoms to arrival at the hospital with PPCI (24.4 h ± 36.5 h) is twice as long as recommended in patients using Brazil’s Unified Health System (SUS, acronym in Portuguese). While the rates of fibrinolytic use are low in both public and private services, they are even lower in SUS patients, and 30-day mortality was also higher in the SUS (11.9%) when compared to patients in the private service (5.9%). These data can be even worse when comparing the health regions of Sergipe. ^5^

In addition, based on the SUS organizational principles, the state is divided into 7 health regions; however, despite the division, all the hospitals with a hemodynamics service are located in a single health region. Moreover, to make matters worse, only one of these hospitals is a cardiology reference for SUS users, and it is not open-doors.

In this context, this study aims to assess the possible differences regarding access to reperfusion therapies and mortality in patients with STEMI who are treated exclusively by the SUS, between the different health regions of Sergipe.

## Material and methods

This is a cross-sectional study with a quantitative approach, conducted with data obtained between December 2014 and March 2018, using the database of the VICTIM Register (Via Crucis for the Treatment of Myocardial Infarction). This study was approved by the Ethics and Research Committee of the Federal University of Sergipe, under opinion number 483,749. The data were collected at the only hospital in the state where primary PPCI is made available by the SUS. This hospital, in turn, does not have an open-door system, that is, patients must be referred from other health services with confirmed diagnosis of STEMI.

Collection was carried out by the researchers using their own research instrument, the case report form (CRF), composed of sociodemographic variables, clinical conditions at hospitalization, data referring to the time and path traveled from the onset of symptoms to the care provided in a specialized hospital, angiography procedure, and evolution of the patients during hospitalization after the acute myocardial infarction. The information was collected through interviews with patients or companions and data from medical records.

Patients of both sexes, over 18 years old, with onset of symptoms within the territory of Sergipe, and with diagnosis of STEMI confirmed by the electrocardiogram, according to the defining criteria proposed by the V Guidelines of the Brazilian Society of Cardiology ^6^ were included, provided that they received care exclusively from the SUS and signed the Free and Informed Consent Form.

The following exclusion criteria were applied: died before the interview; refused to participate in the research; presented onset of symptoms outside the territory of Sergipe; received care in a private network; did not characterize the Via Crucis, that is, those patients who did not travel the path from the onset of the symptoms to the arrival at the hospital with the capacity to perform PPCI because they had already presented STEMI within the hospital; those whose acute STEMI event was characterized as reinfarction (occurring within 28 days of the incident infarction); had a change in diagnosis during hospitalization; and assisted by agreement in a philanthropic hospital.

Once the study inclusion criteria were met, the patients were allocated consecutively. For analysis, patients with STEMI were divided into 7 groups, from the health region where onset of symptoms occurred, namely: 1. Aracaju, 2. Itabaiana, 3. Estância, 4. Lagarto, 5. Nossa Senhora do Socorro (Socorro), 6. Nossa Senhora da Glória (Glória), and 7. Propriá. These health regions were defined according to Deliberation No. 065/2012, of April 18 ^th^ , 2012, which ratifies the division of the state territory of Sergipe into 7 health regions, determining the municipalities that make up each region ( Figure 1 ). Based on the last census conducted by the Brazilian Institute of Geography and Statistics, the state of Sergipe and its 75 municipalities have an estimated population of just over 2 million inhabitants, ^7^ who are divided, in the perspective of the SUS, into these 7 health regions. ^2^

**Figure 1:** – Map of Sergipe and its health regions. Source: state health secretariat (2016)

### Statistical analysis

The categorical variables were described using absolute and relative frequency. The associations were tested using the chi-square test with Monte-Carlo simulations. The multiple comparisons for the proportions were tested using the Z test with Bonferroni correction. The continuous variables were described as median and interquartile range due to their non-adherence to the normal distribution assessed by the Shapiro-Wilk test. The differences in the measures of central tendency were tested using the Kruskal-Wallis test. The multiple comparisons for the measures of central tendency were tested by the Kruskal-Wallis test with Bonferroni correction. The gross and adjusted odds ratios were estimated for overall 30-day mortality through logistic regression. The significance level adopted was 5%, and the software used was R Core Team 2019.

## Results

### Sociodemographic Profile

A total of 844 patients were analyzed, 294 (34.8%) of whom were from the Aracaju health region, 102 (12.1%) from the Itabaiana region, 119 (14.1%) from the Estancia region, 122 (14.5%) from the Lagarto region, 119 (14.1%) from the Socorro region, 41 (4.85%) from the Glória region, and 47 (5.6%) from the Propriá region.

The total median age was 61 years old; among the regions, Estância had a significantly higher median age and the Glória region had the lowest. In all regions, there was prevalence of male patients and non-white ethnicity (70%), with a difference between the Socorro region when compared to Lagarto or Glória (p = 0.02) ( Table 1 ).

**Table 1:** – Sociodemographic characteristics of patients with STEMI by health region*

Demography	All	Aracaju	Itabaiana	Estância	Lagarto	Socorro	Glória	Propriá	p value
	(n=844)	(n=294)	(n=102)	(n=119)	(n=122)	(n=119)	(n=41)	(n=47)	
Age - years old*	61 (52-70)	59 (50-68)	63,5 (50.75-71)	66 (55-74)§//	65 (57-73)§//	60 (51-67)	58 (51,5-69.5)	60 (54-67)	<0.001
**Sex n (%)**									
Male	561 (66.5)	199 (67.7)	77 (75.5)	69 (58.0)	83 (68.0)	72 (60.5)	28 (68.3)	33 (70.2)	0.118
Female	283 (33.5)	95 (32.3)	25 (24.5)	50 (42.0)	39 (32.0)	47 (39.5)	13 (31.7)	14 (29.8)	
**Social class† n (%)**									
A	3 (0.4)	3 (1.1)	0 (0)	0 (0)	0 (0)	0 (0)	0 (0)	0 (0)	0.025
B	17 (2.2)	12 (4.3)	1 (1.1)	1 (0.9)	2 (1.8)	0 (0)	1 (2.9)	0 (0)	
C	74 (9.4)	35 (12.5)	9 (9.8)	7 (6.3)	7 (6.2)	11 (9.9)	1 (2.9)	4 (8.9)	
D	240 (30.5)	97 (34.6)	26 (28.3)	30 (27)	26 (23)	39 (35.1)	12 (34.3)	10 (22.2)	
E	453 (57.6)	133 (47.5)	56 (60.9)	73 (65.8)§	78 (69)§	61 (55)	21 (60)	31 (68.9)	
**Ethnicity n (%)**									
White	248 (30)	85 (29.4)	36 (35.3)	31 (27.7)	45 (36.9)//	21 (18.3)	17 (42.5)//	13 (28.3)	0.020
Non-white	578 (70)	204 (70.6)	66 (64.7)	81 (72.3)	77 (63.1)//	94 (81.7)	23 (57.5)//	33 (71.7)	
**Schooling n (%)**									
Never studied	225 (26.7)	49 (16.7)	38 (37.3)§	41 (34.5)§	50 (41)§¶	28 (23.5)	12 (29.3)	7 (14.9) ‡	<0.001
Elementary school	457 (54.1)	153 (52)	48 (47.1)	70 (58.8)	61 (50)	67 (56.3)	24 (58.5)	34 (72.3)	
High school	121 (14.3)	69 (23.5)	11 (10.8)	7 (5.9)§	8 (6.6)§	19 (16)	4 (9.8)	3 (6.4)	
Higher education	32 (3.8)	20 (6.8)	3 (2.9)	1 (0.8)	3 (2.5)	3 (2.5)	0 (0)	2 (4.3)	
Graduate degree	9 (1.1)	3 (1)	2 (2)	0 (0)	0 (0)	2 (1.7)	1 (2.4)	1 (2.1)	
**Marital Status n (%)**									
Single	115 (13.6)	45 (15.3)	10 (9.8)	15 (12.6)	18 (14.8)	18 (15.1)	4 (9.8)	5 (10.6)	0.050
Married	415 (49.2)	139 (47.3)	52 (51)	51 (42.9)	72 (59)	53 (44.5)	23 (56.1)	25 (53.2)	
Lives with a partner	130 (15.4)	42 (14.3)	13 (12.7)	26 (21.8)	15 (12.3)	20 (16.8)	9 (22)	5 (10.6)	
Divorced	73 (8.6)	33 (11.2)	10 (9.8)	4 (3.4)	7 (5.7)	8 (6.7)	4 (9.8)	7 (14.9)	
Widowed	111 (13.2)	35 (11.9)	17 (16.7)	23 (19.3)	10 (8.2)	20 (16.8)	1 (2.4)	5 (10.6)	

* The values are median and interquartile range. Social class (Brazilian Institute of Geography and Statistics) – A: > 20 times minimum wage, B: 10 to 20 times minimum wage, C: 4 to 10 times minimum wage, D: 2 to 4 times minimum wage, E: ≤ 2 times minimum wage. ‡ p < 0.05 when compared to Lagarto. § p < 0.05 when compared to Aracaju. // p < 0.05 when compared to Socorro ¶ p < 0.05 when compared to Propriá.

#### Clinical features

Among the risk factors, diabetes mellitus was the only one that presented significant variability, ranging from 17.1% in Glória to 42.6% in Propriá (p = 0.026). The other risk factors showed similar prevalence values between the groups ( Table 2 ).

**Table 2:** – Clinical characteristics of the patients with STEMI by health regions*

Clinical characteristics	All		Aracaju	Itabaiana	Estância	Lagarto	Socorro	Glória	Propriá	p value
	(n=844)		(n=294)	(n=102)	(n=119)	(n=122)		(n=41)	(n=47)	
**Cardiovascular risk n (%)**							**(n=119)**			
Hypertension	530	(62.8)	176 (59.9)	61 (59.8)	72 (60.5)	76 (62.3)	86 (72.3)	26 (63.4)	33 (70.2)	0.282
Diabetes	275	(32.6)	104 (35.4)	24 (23.5)	46 (38.7)	35 (28.7)	39 (32.8)	7 (17.1)	20 (42.6)	0.026
Dyslipidemia	314	(37.2)	116 (39.5)	33 (32.4)	46 (38.7)	45 (36.9)	39 (32.8)	15 (36.6)	20 (42.6)	0.762
Smoking	282	(33.4)	102 (34.7)	37 (36.3)	32 (26.9)	32 (26.2)	45 (37.8)	20 (48.8)	14 (29.8)	0.078
Number of risk factors n (%)										
0	100	(11.8)	38 (12.9)	14 (13.7)	15 (12.6)	20 (16.4)	6 (5)	3 (7.3)	4 (8.5)	0.255
1	279	(33.1)	90 (30.6)	36 (35.3)	39 (32.8)	44 (36.1)	42 (35.3)	15 (36.6)	13 (27.7)	
2	297	(35.2)	100 (34)	38 (37.3)	40 (33.6)	35 (28.7)	50 (42)	18 (43.9)	16 (34)	
≥ 3	168	(19.9)	66 (22.4)	14 (13.7)	25 (21)	23 (18.9)	21 (17.6)	5 (12.2)	14 (29.8)	
**Systemic blood pressure**										
Systolic blood pressure	140 (123-160)		145 (130-160.75)	140 (123.7-157.5)	135 (120-160)	140 (120,75-157)	140 (120.75-160)	130 (120-147)¶	140 (120-160)¶	0.016
Diastolic blood pressure	83 (75-96)		87,5 (78-99.25)	87 (76.75-93)	80 (70-99)	80 (71,75-90)	87 (77-98.5)	80(70-87.5)	80 (71-90)	0.007
**Killip‡**										
I	716	(85.3)	245 (83.9)	92 (92)	101 (84.9)	102 (84.3)	104 (87.4)	35 (85.4)	37 (78.7)	0.557
II	92 (11)		35 (12)	7 (7)	14 (11.8)	14 (11.6)	10 (8.4)	5 (12.2)	7 (14.9)	
III	18	(2.1)	7 (2.4)	1 (1)	3 (2.5)	2 (1.7)	1 (0.8)	1 (2.4)	3 (6.4)	
IV	13	(1.5)	5 (1.7)	0 (0)	1 (0.8)	3 (2.5)	4 (3.4)	0 (0)	0 (0)	
**GRACE score§**										
< 140 (low risk)	392	(50.2)	154 (58.1)#	41 (45.1)	51 (45.1)	42 (35.6)	63 (56.3)#	23 (56.1)	18 (43.9)	0.001
> 140 (high risk)	389	(49.8)	111 (41.9)#	50 (54.9)	62 (54.9)	76 (64.4)	49 (43.8)#	18 (43.9)	23 (56.1)	
**Electrocardiogram//**										
Previous	527	(62.5)	198 (67.3)	63 (61.8)	71 (59.7)	74 (60.7)	68 (57.6)	22 (53.7)	31 (66)	0.384
Non-previous	316	(37.5)	96 (32.7)	39 (38.2)	48 (40.3)	48 (39.3)	50 (42.4)	19 (46.3)	16 (34)	

* The values are median and interquartile range. † Value of the first record (in millimeters of mercury) documented in the medical record after hospital admission. Upon admission - Class I: absence of rales on the pulmonary fields and absence of a third sound, Class II: rales in more than 50% of the areas of the lung or presence of a third sound, Class III: rales in more than 50% of the lung fields, Class IV: Shock. Ranging from 0 to 263 calculated using the data on age, heart rate, systolic blood pressure, creatinine and Killip recorded at hospital admission. // Previous: on V1-V4 referrals, non-previous: on any of the V1-V4 referrals ¶ p < 0.05 when compared to Aracaju. # p < 0.05 when compared to Lagarto.

Regarding the values of systemic blood pressure upon admission to the hospital with PPCI ( Table 2 ), systolic blood pressure had a total median of 140 mmHg, presenting the highest value in the Aracaju region and the lowest in Nossa Senhora da Glória, with differences when compared to each other (p = 0.016). Diastolic blood pressure, on the other hand, reached a total median of 83 mmHg, with the highest median in Aracaju, Nossa Senhora do Socorro, and Itabaiana, and the lowest median in Nossa Senhora da Glória, Estância, Lagarto, and Propriá (p = 0.007).

The Lagarto region showed the highest rate of patients considered to be at high risk of mortality by the GRACE score (64.4%) ( Table 2 ), while Aracaju had the lowest (41.9%), and it possible to observe a difference when comparing the Aracaju and Nossa Senhora da Glória regions in relation to Lagarto (p = 0.001). Of the total number of patients, 85.3% had Killip I, and 62.5% of the STEMI were of the anterior wall; this behavior pattern was repeated across the regions.

#### Coronary reperfusion

The total primary PPCI rate was 45.8%, with the Aracaju region having the highest (51.9%) and Glória the lowest (17.1%), with a difference when compared to Aracaju or Itabaiana (p = 0.03). Of the total, 25.1% of the study patients did not undergo PPCI, and the total rate of fibrinolytic use was 2.6% ( Table 3 ).

**Table 3:** – Procedures performed on patients with STEMI by health regions*

Procedure‡	All	Aracaju	Itabaiana	Estância	Lagarto	Socorro	Glória	Propriá	p value
	(n=844)	(n=294)	(n=102)	(n=119)	(n=122)	(n=119)	(n=41)	(n=47)	
Fibrinolytic n (%)	22 (2.6)	3 (1)	3 (2.9)	5 (4.2)	5 (4.1)	4 (3.4)	1 (2.4)	1 (2.1)	0.477
Primary PCI n (%)	386 (45.8)	151 (51.4)†	51 (50)†	55 (46.6)†	53 (43.4)	51 (42.9)	7 (17.1)	18 (38.3)	0.003
Non-primary PCI n (%)	275 (32.6)	84 (28.6)	31 (30.4)	44 (37)	37 (30.3)	43 (36.1)	21 (51.2)	15 (31.9)	0.092
Did not perform PCI n (%)	212 (25.1)	67 (22.8)	23 (22.5)	29 (24.6)	36 (29.5)	29 (24.4)	13 (31.7)	15 (31.9)	0.595
Myocardial revascularization surgery	24 (2.8)	7 (2.4)	5 (4.9)	3 (2.5)	2 (1.6)	2 (1.7)	3 (7.3)	2 (4.3)	0.383

*‡ PCI: percutaneous coronary intervention. † p < 0.05 when compared to Glória.*

The mean time between the onset of symptoms and arrival at the hospital with PPCI was 21 hours and 55 minutes with a median of 10 hours and 22 minutes (6 hours and 30 minutes to 22 hours and 52 minutes), with Glória showing the longest delay, and Aracaju the shortest. A statistical difference was recorded when the Glória and Socorro regions were compared in relation to Aracaju (p = 0.001). Of the periods that make up this entire time interval, the time elapsed between arrival at a hospital without PPCI and arrival at a hospital with PPCI was the most impactful, recording a median time of 7 hours and 37 minutes. In this regard, the region with the longest delay was Glória, and the one with the lowest was Aracaju; it was possible to observe differences when comparing the Lagarto and Glória regions with Aracaju ( Table 4 ).

**Table 4:** – Time and geographical course between onset of symptoms and arrival at the hospital with PPCI by health region*

	All	Aracaju	Itabaiana	Estância	Lagarto	Socorro	Glória	Propriá	p value
	(n=844)	(n=294)	(n=102)	(n=119)	(n=122)	(n=119)	(n=41)	(n=47)	
Timeline *†									
Time A	30’ (10’-2h)	30’ (10’-2h37’)	30’ (15’-2h)	30’ (10’-2h)	30’ (15’-2h)	30’ (10’-2h)	30’ (17’-4h)	1h (10’-2h)	0,808
Time B	30’ (20’-1h)	30’ (15’-48’)	30’ (20’-1h)	30’ (20’-1h)	30’ (15’-50’)	30’ (18’-1h)	30’ (20’-1h)	30’ (20’-1h)	0,038
Time C	7h37’ (4h51’-18h)	6h30’ (3h30’-15h40’)	7h30’ (4h53’-13h)	7h50’ (5h20’-11h16’)	8h34’ (5h28’-21h25’) ‡	8h17’ (4h40’-28h15’)	12h40’ (6h45’-26h56’)‡	8h57’ (6h35’-14h30’)	<0,001
Time D	10h22’ (6h30’-22h52’)	9h (5h7’-21h32’)	10h (6h9’-15h47’)	10h30’ (7h10’-19h45’)	11h31’ (7h3’-25h20’)	12h40’ (6h30’-32h)‡	16h (8h30’-32h01’)‡	12h27’ (7h50’-18h08’)	0,001
**No. of units visited**									
0	14 (1,7)	9 (3,1)	1 (1)	1 (0,8)	2 (1,6)	1 (0,8)	0 (0)	0 (0)	0,483
1	685 (81,2)	248 (84,4)	85 (83,3)	91 (76,5)	100 (82)	93 (78,2)	31 (75,6)	37 (78,7)	
2	125 (14,8)	32 (10,9)	14 (13,7)	24 (20,2)	17 (13,9)	20 (16,8)	9 (22)	9 (19,1)	
≥ 3	20 (2,4)	5 (1,7)	2 (2)	3 (2,5)	3 (2,5)	5 (4,2)	1 (2,4)	1 (2,1)	

* The values are median and interquartile range. Time A: from onset of symptoms to decision to call for help, Time B: decision to call for help to the hospital without PPCI, Time C: time to go from the hospital without PPCI to the hospital with PPCI, Time D: onset of symptoms at the hospital with PPCI. p < 0.05 when compared to Aracaju.

Regarding the number of institutions visited before the hospital with PPCI, the vast majority of the patients (81.2%) went through at least one institution before the hospital with PPCI. Nearly 2.4% of the patients went to at least three institutions before the hospital with PPCI, while only 1.7% had direct access to this hospital ( Table 4 ).

#### Mortality

When assessing 30-day mortality ( Table 5 ), it was observed that the Estância region had the highest rate (18.6%), and Nossa Senhora da Glória had the lowest (7.5%) (p = 0.03). When adjusted for age and sex, adopting the Aracaju region as a control group, no statistical differences were observed.

**Table 5:** – Odds ratio for 30-day mortality in patients with STEMI by health regions, adjusted for age and sex

Health regions	Mortality N (%)	Non-adjusted mortality OR (95%CI)	Adjusted mortality OR (95%CI)	p value
Aracaju*	27 (9.4) 27 (9.4)	1	1	
Itabaiana	9 (9.2)	0.98 (0,44-2.16)	0.93 (0.32-2.70)	0.900
Estância	22 (18.6)	2.22 (1.20-4.08)	1.70 (0.74-3.92)	0.214
Lagarto	22 (18.5)	2.19 (1.19-4.03)	2.07 (0.93-4.61)	0.074
Socorro	15 (12.7)	1.41 (0.72-2.75)	2.04 (0.86-4.87)	0.106
Glória	3 (7.5)	0.78 (0.23-4.68)	0.66 (0.13-3.48)	0.625
Propriá	8 (17)	1.98 (0.84-4.68)	1.93 (0.61-6.11)	0.263

*Represents the control group.

## Discussion

Three main findings expressively marked the results of this study. The first points to major delays in the arrival of patients with STEMI at the hospital with PPCI, regardless of the onset of symptoms. The second testifies to disparities in the use of reperfusion therapies between patients in the different health regions. The third shows the regional influence on mortality. These findings demonstrate that there is an urgent need to improve quality of care for patients with STEMI throughout the state of Sergipe.

Despite greater knowledge about the therapeutic goals in the management of STEMI, the reproducibility of these targets is still a difficult task, especially in the field of public health in Brazil. ^8 , 9^ Patients with onset of STEMI in the state of Sergipe have a 30-day mortality rate far from what is considered desirable. A report by the European Society of Cardiology, including several countries, showed in-hospital mortality ranging from 3.1% to 6.1%. ^10^ A French trial, carried out between 1995 and 2010, found a reduction in 30-day mortality from 13.7% to 4.4%. This decrease is due to multiple factors within care for patients with STEMI, such as the increase in the number of mobile intensive care units, an increase in the number of public information campaigns on the symptoms related to the disease, and lesser delays in both arrival at a qualified hospital and in the decision to seek help. ^11^

Concomitantly, both regions with the worst rates presented the highest mean age groups. Despite the reduction in mortality due to acute coronary syndromes at all ages, it is known that older patients have worse prognosis compared to younger ones, due both to the fact that they have a higher number of comorbidities and to the lesser use of reperfusion and medication therapies. ^3 , 12 , 13^

Before arriving at the hospital with the capacity to perform PPCI, some patients had to go through at least one health unit lacking this service until they were transferred to the specialized hospital. These data were also analyzed in this study and the result obtained was that most of the patients went to a hospital before arriving at the hospital with the capacity to perform PPCI, a fact that was already expected, since the only public hospital with capacity for performing PPCI does not offer open-door service. Some patients who have direct access to the hospital with PPCI were regulated and referred by the Mobile Emergency Care Service.

The period between the onset of symptoms and access to a hemodynamics service plays a decisive role in the patients’ prognosis. ^14^ In Sergipe, in this period the mean time spent was 21 hours and 55 minutes with a median of 10 hours and 22 minutes (6 hours and 30 minutes to 22 hours and 52 minutes), bordering twice the 12-hour window established by the national and international guidelines. Evaluating from a regional perspective, this mean interval reached values of 26 hours and 24 minutes and 26 hours and 10 minutes in the Socorro and Glória regions, respectively, and they were shorter in Estância, with 16 hours and 22 minutes. However, despite this discrepancy, it was only possible to observe statistical differences when comparing Socorro and Glória to Aracaju. It is worth highlighting that the municipality farthest from the capital is nearly 3 hours away.

In the state of Sergipe, the time interval experienced by patients with STEMI from the onset of symptoms to access to a qualified hospital suffers a huge impact from the hospital interim period, constituting approximately 87% of the entire process. From this perspective, these results do not correspond to the geographic reality of Sergipe, which is considered the smallest state in Brazil, where it takes around 4 hours to go from one end of the state to the other by car. In addition to that, the Socorro region, despite its relative proximity to the Aracaju region compared to the others, paradoxically presents the largest of these delays, with 23 hours and 15 minutes.

Several elements can be linked to the prolongation of this interval, ranging from delays in disease diagnosis to inefficiency in the transportation means between the assisting institutions. ^1 , 15^ A piece of data that deserves to be highlighted is the impossibility of referring patients directly to the qualified hospital. In Sergipe, the only hospital capable of offering definitive treatment to SUS patients with STEMI only receives patients through referrals made by another institution, as long as they are already diagnosed.

A study carried out in the state of North Carolina, in the period between 2008 and 2010, evaluated 1,288 patients diagnosed with STEMI, dividing them into two groups, those who were transferred directly to hospitals with PPCI regardless of the distance and those who were transported to the nearest hospital without PPCI. In this comparison, 46.5% of the patients directly transferred to a center with a hemodynamics service arrived within 90 minutes after the first medical contact, while, in the other group, only 21.5% of the patients arrived at a hemodynamics center within 120 minutes after the first medical contact. ^16^

Considering the major difficulty of access in the only hospital with the capacity to perform PPCI, less than half of the patients arrive within the 12-hour window from the onset of symptoms; the expressive underuse of fibrinolytics in the state of Sergipe can be considered an indicator of a very deficient care practice contributing to the high mortality observed in our records. The mean rate of fibrinolytic use of 2.6% differs greatly from other national and international records, ^17 , 18^ and, in the scenario of difficult access to the only hospital capable of performing PPCI, it unequivocally exposes the important weakness and inefficiency of STEMI care in our state. ^4^ Our findings could be even more critical, if the population studied was composed of all patients with STEMI in the state, that is, those who stayed in primary and secondary hospitals, without access to the tertiary hospital. Incorporating the network of regional hospitals with the capacity to perform thrombolysis in the scenario may increase the total rate of patients who are reperfused and reduce mortality in this setting.

Although primary angioplasty is the treatment of choice for these patients, in this study, only 45.8% of the patients underwent this therapy, reaching more striking values from the perspective of the Glória region, with 17.1%. These rates are below those found in other studies. ^19 - 22^ Despite the variability across the regions, it was only possible to observe a statistical difference when comparing the Lagarto region to Aracaju.

The discrepancies found both in the PPCI rate and in the time to access this method are justified by the same failures in the health care network. A study conducted in the United Kingdom showed that, in the second half of 2011, 94% of the patients with STEMI were treated by PPCI, a significant increase when compared to the rate of 46% in 2008. Among the elements responsible for this progression are the following: direct transportation of patients to centers with PPCI, professional training for pre-hospital diagnosis, data collection regarding quality of care, and, finally, creation of national policies in order to facilitate access to the health care network. ^23^

Another study carried out in the United Kingdom using data from England and Wales, with a total of 228 hospitals and a sample of 34,722 patients with STEMI, showed that the use of aspirin at admission and thrombolysis outside the hospital are the strongest predictors of in-hospital survival. In addition to that, factors such as heart rate and systolic blood pressure at admission also impact 30-day mortality due to STEMI. ^3^

## Limitations

In view of the precariousness of well-documented records in some of the regions, data acquisition was supplemented through interviews, and part of these were self-reported, which can lead to inaccuracies in the time measures. It is also worth noting that many SUS users are unaware of their previous health status, making it difficult to accurately measure the prevalence of comorbidities; this fact hinders adjustment of the risk across the different populations of the regions under study. Finally, this study restricted data collection to the hospital with PPCI, as it is the only reference in the treatment of STEMI in the state. This fact limits the results to the patients who had access to the reference center. However, we emphasize that, by limiting the population to the reference center, the observed scenario represents the best quality of care provided by the SUS in the state of Sergipe.

## Conclusion

The VICTIM Register identified a glaring lack of sufficient access to PPCI in the state, recording a primary time window of access almost double that which is considered borderline, which was even worse in some sub-regions. In addition, there is marked underuse of fibrinolytics, as an alternative reperfusion therapy for STEMI, in all regions. High mortality rates persist, despite the therapeutic advances in cardiovascular science in the era of myocardial reperfusion. Together, our data demonstrate great inefficiency of the SUS in terms of assistance to patients with STEMI in the state of Sergipe. Our results should be investigated in other states and regions of the country to assess whether the precarious indicators herein observed are peculiar to Sergipe and the inserted region, or whether they represent “standard” care in the Brazilian public health system.
